# *Salmonella enterica* Serovar Typhi in Bangladesh: Exploration of Genomic Diversity and Antimicrobial Resistance

**DOI:** 10.1128/mBio.02112-18

**Published:** 2018-11-13

**Authors:** Arif M. Tanmoy, Emilie Westeel, Katrien De Bruyne, Johan Goris, Alain Rajoharison, Mohammad S. I. Sajib, Alex van Belkum, Samir K. Saha, Florence Komurian-Pradel, Hubert P. Endtz

**Affiliations:** a Department of Medical Microbiology and Infectious Diseases, Erasmus University Medical Center, Rotterdam, the Netherlands; b Fondation Mérieux and Centre International de Recherche en Infectiologie (CIRI), INSERM, Lyon, France; c Child Health Research Foundation, Department of Microbiology, Dhaka Shishu Hospital, Dhaka, Bangladesh; d Applied Maths, Sint-Martens-Latem, Belgium; e Data Analytics Unit, bioMérieux, La Balme Les Grottes, France; f Bangladesh Institute of Child Health, Dhaka Shishu Hospital, Dhaka, Bangladesh; Emory University; International Centre for Diarrhoeal Disease Research, Bangladesh; Centers for Disease Control & Prevention

**Keywords:** Bangladesh, *Salmonella* Typhi, antibiotic resistance, genomics

## Abstract

Typhoid fever, caused by Salmonella enterica serovar Typhi, is a global public health concern due to increasing antimicrobial resistance (AMR). Characterization of *S*. Typhi genomes for AMR and the evolution of different lineages, especially in countries where typhoid fever is endemic such as Bangladesh, will help public health professionals to better design and implement appropriate preventive measures. We studied whole-genome sequences (WGS) of 536 *S*. Typhi isolates collected in Bangladesh during 1999 to 2013 and compared those sequences with data from a recent outbreak in Pakistan reported previously by E. J. Klemm, S. Shakoor, A. J. Page, F. N. Qamar, et al. (mBio 9:e00105-18, 2018, https://doi.org/10.1128/mBio.00105-18), and a laboratory surveillance in Nepal reported previously by C. D. Britto, Z. A. Dyson, S. Duchene, M. J. Carter, et al. [PLoS Negl. Trop. Dis. 12(4):e0006408, 2018, https://doi.org/10.1371/journal.pntd.0006408]. WGS had high sensitivity and specificity for prediction of ampicillin, chloramphenicol, co-trimoxazole, and ceftriaxone AMR phenotypes but needs further improvement for prediction of ciprofloxacin resistance. We detected a new local lineage of genotype 4.3.1 (named lineage Bd) which recently diverged into a sublineage (named Bdq) containing *qnr* genes associated with high-level ciprofloxacin resistance. We found a ceftriaxone-resistant isolate with the *bla*_CTX-M-15_ gene and a genotype distinct from the genotypes of extensively drug-resistant (XDR) isolates from Pakistan. This result suggests a different source and geographical origin of AMR. Genotype 4.3.1 was dominant in all three countries but formed country-specific clusters in the maximum likelihood phylogenetic tree. Thus, multiple independent genetic events leading to ciprofloxacin and ceftriaxone resistance took place in these neighboring regions of Pakistan, Nepal, and Bangladesh. These independent mutational events may enhance the risk of global spread of these highly resistant clones. A short-term global intervention plan is urgently needed.

## Abstract (precis)

Typhoid fever, caused by Salmonella enterica serovar Typhi, is responsible for an estimated burden of approximately 17 million new episodes per year worldwide. Adequate and timely antimicrobial treatment invariably cures typhoid fever. The increasing antimicrobial resistance (AMR) of *S*. Typhi severely limits the treatment options. We studied whole-genome sequences (WGS) of 536 *S*. Typhi isolates collected in Bangladesh between 1999 and 2013 and compared those sequences with data from a recent outbreak in Pakistan and a laboratory surveillance in Nepal. The analysis suggests that multiple ancestral origins of resistance against ciprofloxacin and ceftriaxone are present in three countries. Such independent genetic events and subsequent dissemination could enhance the risk of a rapid global spread of these highly resistant clones. Given the current treatment challenges, vaccination seems to be the most appropriate short-term intervention to reduce the disease burden of typhoid fever at a time of increasing AMR.

## INTRODUCTION

Typhoid fever is a life-threatening infectious disease caused by Salmonella enterica serovar Typhi. *S*. Typhi colonizes only humans, is transmitted through the fecal-oral route, and is endemic in tropical countries, especially in Africa and South and Southeast Asia. Worldwide, approximately 17 million people are infected every year by this pathogen (1–4). Though the mortality rate remains low (<1%), 1 in 20 to 25 cases experiences residual disability (5).

Adequate and timely antimicrobial treatment invariably cures typhoid fever. However, the increasing antimicrobial resistance (AMR) of *S*. Typhi limits the treatment options. In spite of suggested regional decreases in the levels of antibiotic resistance (6–8), the first cases of *S*. Typhi isolates showing multidrug resistance (MDR) (defined as co-occurring resistance to ampicillin [amp], chloramphenicol [chl], and co-trimoxazole [sxt]) were reported in the early 1970s (9, 10). Ciprofloxacin (cip) resistance first emerged in the early 1990s. At present, over 90% of clinical isolates from regions of endemicity show reduced susceptibility to ciprofloxacin (6, 7, 11). These events shifted the first-line and empirical treatments to other classes of antimicrobial agents, such as ceftriaxone (cro) and azithromycin. Alarmingly, reports of resistance against these agents have now been published (6, 12–18). Moreover, a recent report from Pakistan described the first large-scale outbreak of an *S*. Typhi clone that is extensively drug resistant (XDR; defined as MDR plus resistance to ciprofloxacin and ceftriaxone) (12).

Whole-genome sequence (WGS)-based approaches using next-generation sequencing (NGS) have become effective tools for the study of genetic diversity and prediction of resistance phenotypes (12, 19–25). Several studies have correlated WGS data with various resistance phenotypes in *S*. Typhi (12, 20, 25, 26). However, most of these studies involved small numbers of isolates from multiple countries, isolates from single outbreaks, or clusters of travel-related typhoid cases, which do not accurately represent the situation in countries where typhoid fever is endemic over longer periods of time (12, 20, 25–29). These shortcomings limit our overall understanding fo the dynamics of typhoid fever in regions of endemicity, especially in South Asia, where the disease burden is high.

We generated a WGS data set of 536 *S*. Typhi strains, which were mostly isolated from the blood of pediatric patients in Bangladesh over a period of 15 years (1999 to 2013). In this study, we explored the phenotypic and genotypic diversity of these isolates using whole-genome single nucleotide polymorphism (wgSNP) analysis, classical multilocus sequence typing (MLST), and core genome MLST (cgMLST). We also examined the phylogenetic relationships between these isolates and compared the results with two published data sets from two neighboring countries, representing a hospital-based surveillance study in Nepal and an outbreak during 2016 to 2017 in Pakistan (12, 29). Additionally, we investigated the utility of NGS data for the prediction of phenotypic resistance to multiple antibiotics. We focused on the genes involved in MDR and mutations in the DNA gyrase (*gyrA* and *gyrB*) and topoisomerase IV (*parC* and *parE*) enzymes that lead to ciprofloxacin resistance.

## RESULTS

### Genotypic diversity of *S*. Typhi in Bangladesh.

**FIG 1:**
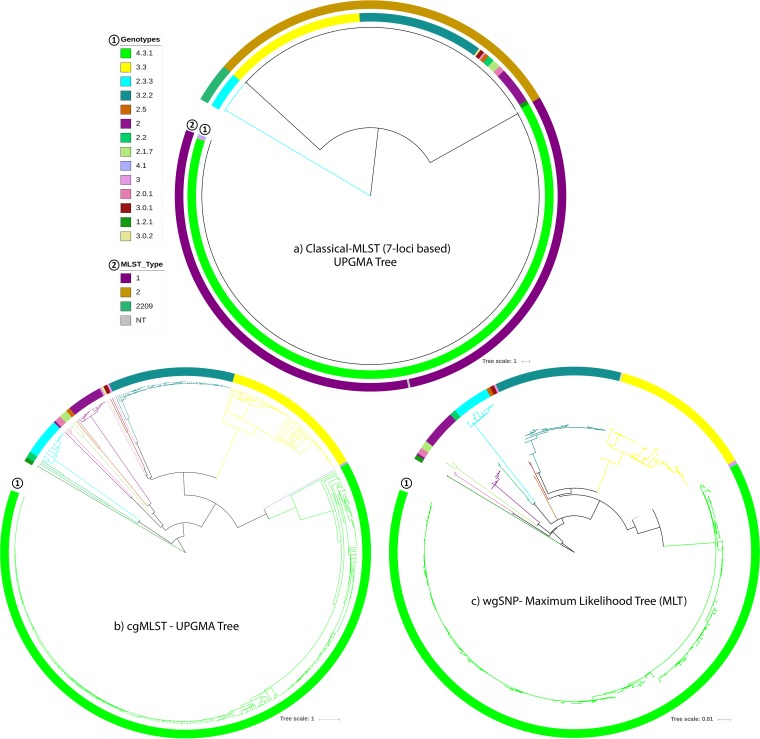
Genomic diversity and phylogenetic relationships among *S*. Typhi isolates from Bangladesh. (a and b) UPGMA trees constructed on the basis of (a) Classical MLST (7-locus-based) results in comparison with the genotypes and (b) core-genome MLST (cgMLST) results. (c) Maximum likelihood tree (MLT) constructed on the basis of results of whole-genome SNP (wgSNP) analyses. All phylogenetic trees are colored according to genotypes.

Among the 539 strains presumptively identified as *S*. Typhi, 536 (99%) were confirmed to be *S*. Typhi by WGS-based serotyping and were analyzed further. A total of 61% (329/536) of them were from hospitalized patients. Genotype 4.3.1 was dominant (65%; 350/536), followed by genotype 3.3 (13%; 69/536), genotype 3.2.2 (11%; 61/536), and 12 other genotypes (Table 1). Classical MLST analysis revealed the presence of only three different sequence types (ST) among our isolates, namely, ST1 (*n* = 351), ST2 (*n* = 166), and ST2209 (*n* = 18) (Fig. 1a), all of which had similar ratios of hospitalized and outpatient cases (∼60% versus ∼40%). Overall, 99% of the ST1 strains had the 4.3.1 genotype (349/351), while all of the ST2209 strains had genotype 2.3.3 (18/18; 100%). Other genotypes were within ST2 (Fig. 1a). One isolate was nontypeable (NT) by MLST analysis. With respect to the haplotyping scheme, haplotype 58 (H58) was dominant (65%; 350/536), followed by H1 (130/536; 24%) (Table 1).

**TABLE 1:** Genotyping and haplotyping results for the 536 *S*. Typhi isolates from Bangladesh based on WGS

Genotype	No. of isolates	% of total	Haplotype
4.3.1	350	65.30	H58
3.3	69	12.87	H1
3.2.2	61	11.38	H1
2	18	3.36	NT
2.3.3	18	3.36	NT
2.1.7	4	0.75	H8
2.0.1	3	0.56	NT
2.2	3	0.56	NT/H39
1.2.1	2	0.37	NA^*a*^
2.5	2	0.37	H55
3	2	0.37	H13
3.0.1	2	0.37	H13
3.0.2	1	0.19	H13
4.1	1	0.19	H52/15/10/67
			
Total	536	100	

a NA, not available.

### Phylogenetic relationships and new lineages.

Genotypes 3.2.2 and 3.3 clustered together in the same classical MLST type (ST2) but formed two distinct subclades in the unweighted pair group method using average linkages (UPGMA) tree based on cgMLST analyses of 3,002 core loci (Fig. 1a and b). Like genotype 2.3.3, genotype 4.3.1 also generated its own subclade in the tree, with the notable presence of multiple subgroups (Fig. 1b). The same UPGMA tree presented a distinct population structure and a similar genotypic differentiation as observed in the maximum likelihood tree (MLT), which was based on 2,328 SNPs from our WGS data compared to the *S*. Typhi CT18 reference genome (Fig. 1c) (30).

Genotype 1.2.1 mapped closest to the root of the MLT; genotype 4.3.1 was the most remote (Fig. 1c). As the dominant genotype, 4.3.1 formed a large subclade, with genotype 4.1 in its primary clade. Another primary clade divided into two major subclades, genotypes 3.3 and 3.2.2, which comprised the second and third most prevalent genotypes in Bangladesh. Two small subclades of genotype 2.3.3 and genotype 2.0 were also present in the MLT, rooting with genotype 2.2 and 2.1.7, respectively.

**FIG 2:**
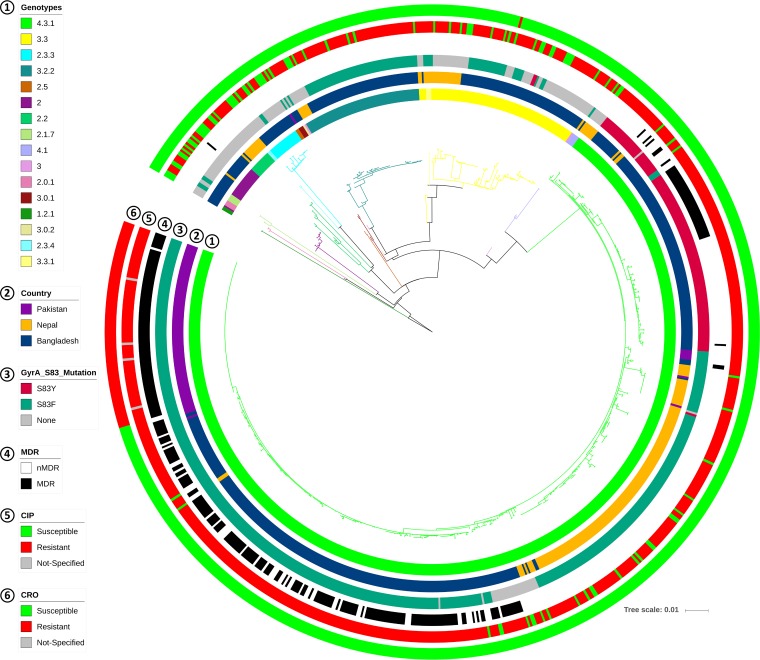
Comparison of Bangladesh isolates with Pakistan and Nepal isolates in a wgSNP-derived MLT. No singleton was considered in the consensus SNP data. The tree is colored by genotype. Different data points, including country, presence of different *gyrA*-83 mutations, MDR, and cip resistance and cro resistance phenotypes are indicated (by colors) in different circles around the tree. nMDR, no multidrug resistance.

**FIG 3:**
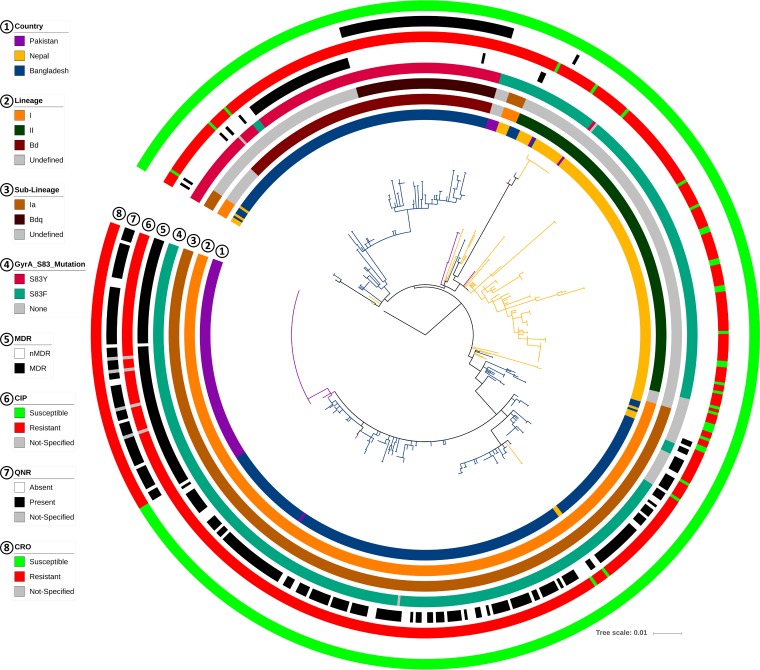
Comparison of genotype 4.3.1 (H58) isolates from Bangladesh, Pakistan, and Nepal in a wgSNP-derived MLT. No singleton was considered in the consensus SNP data. The tree is colored by country. Different data points, including lineage, sublineage (details), presence of different *gyrA*-83 mutations, MDR, cip resistance, presence of *qnr* genes, and cro resistance phenotypes are indicated (by colors) in different circles around the tree.

The comparative MLT created using our data and the strains from neighboring countries showed country-specific clusters inside the dominant 4.3.1 genotype and also in genotypes 3.3 and 3.2.2 (Fig. 2 and 3). This suggests different points of origin for the various lineages of *S*. Typhi in each country. All XDR Pakistani isolates extended into a single branch of the MLT, showing H58 lineage Ia and a minimally divergent pattern (Fig. 3; see also Fig. S1 in the supplemental material), whereas the Nepali strains were dominant in lineage II. The isolates from Bangladesh included only four isolates from lineage II but showed two distinct clusters inside genotype 4.3.1 (H58): lineage Ia (*n* = 223) and a previously nondescribed lineage (*n* = 108; Fig. 3). The latter lineage did not match the SNP definition of H58 lineage I or lineage II, suggesting a previously undetected H58 lineage (genotype 4.3.1). On the basis of the MLT, this nondescribed lineage could have had the same point of origin as lineage I but then clearly followed a different pattern of divergence and formed its own subclade inside genotype 4.3.1 (Fig. 3). This new H58 lineage can be distinguished by the SNPs at nucleotide position 561056 (C→A) and 2849843 (A→C) of the CT18 reference genome (this previously undescribed lineage is referred to as “lineage Bd” in the remainder of the article).

10.1128/mBio.02112-18.1 FIG S1 Comparison of genotype 4.3.1 (H58) isolates from Bangladesh, Pakistan, and Nepal in a wgSNP-derived (with singleton) MLT. (a) MLT with all singletons. (b) Divergence among all XDR isolates from Pakistan. All singletons were considered in the consensus SNP data. The tree is colored by country. Different data points, such as those corresponding to lineages, sublineages (details), the presence of different *gyrA*-83 mutations, MDR, cip resistance, presence of *qnr* genes, and cro resistance phenotypes, are shown (by colors) in different circles around the tree. Download FIG S1, TIF file, 3.4 MB. Copyright © 2018 Tanmoy et al. 2018 Tanmoy et al. This content is distributed under the terms of the Creative Commons Attribution 4.0 International license.

### Resistance phenotypes and genotypes.

On the basis of analyses performed with five different antibiotics—ampicillin (amp), chloramphenicol (chl), co-trimoxazole (sxt), ciprofloxacin (cip), and ceftriaxone (cro)—the 536 *S*. Typhi isolates from Bangladesh were found to harbor 12 different phenotypic resistance profiles (phenotypes; Table 2). Isolates with the “MDR, cip-R” profile (*n* = 202) were most prevalent in our library, followed by “cip-R only” (*n* = 169) and “Susceptible to all” (*n* = 62). A comparison of MDR and ciprofloxacin-resistant isolates with different genotypes is presented in Table 3. The single ceftriaxone-resistant (cro-R) strain (MIC > 32 µg/ml) (susceptible to sxt, chl, and cip) in our library, isolated in 2000, displayed genotype 3.3 (haplotype H1) and contained the *bla*_CTX-M-15_ gene (ceftriaxone resistance). The other resistance genes detected are listed in Table 4, including *bla*_TEM-1B_ (ampicillin resistance); *catA1* (chloramphenicol resistance); *dfrA7*, *sul1*, and *sul2* (co-trimoxazole resistance); and *qnrS1* (ciprofloxacin resistance).

**TABLE 2:** Resistance phenotypes in our library of 536 *S*. Typhi isolates^*a*^

Phenotype	No. of isolates	% of total
MDR, cip-R	202	37.69
MDR	4	0.75
amp-R, sxt-R, cip-R	1	0.19
amp-R, chl-R, cip-R	2	0.37
amp-R, cip-R	53	9.89
amp-R, cro-R	1	0.19
sxt-R, chl-R, cip-R	25	4.66
sxt-R, chl-R	1	0.19
chl-R, cip-R	15	2.80
chl-R only	1	0.19
cip-R only	169	31.53
Susceptible to all	62	11.57
		
Total	536	100

a Five different antibiotics were considered: ampicillin (amp), co-trimoxazole (sxt), chloramphenicol (chl), ciprofloxacin (cip), and ceftriaxone (cro). MDR (multidrug resistance) refers to co-occurring resistance to amp, sxt, and chl. “S” and “R” refer to susceptible and resistant phenotypes, respectively (interpretations according to EUCAST-2018).

**TABLE 3:** Comparison between multidrug resistance (MDR) and ciprofloxacin resistance (cip-R) with genotypes among Bangladesh isolates

Genotype	No. of isolates				
	MDR		cip-R		Total
	Yes	No	Yes	No	
1.2.1	0	2	0	2	2
2	1	17	6	12	18
2.0.1	0	3	3	0	3
2.1.7	0	4	2	2	4
2.2	0	3	2	1	3
2.3.3	0	18	8	10	18
2.5	0	2	0	2	2
3	0	2	1	1	2
3.0.1	0	2	1	1	2
3.0.2	0	1	0	1	1
3.2.2	0	61	52	9	61
3.3	0	69	52	17	69
4.1	0	1	1	0	1
4.3.1	205	145	339	11	350
Total	206	330	467	69	536

**TABLE 4:** List of resistance genes detected in our isolates^*a*^

Resistance gene	Antibiotic class	No. of isolates	% of total	Phenotype	Matched NCBI accession no.
*bla*_TEM-1B_	Beta-lactam	271	50.28	amp-R	JF910132
*bla*_CTX-M-15_	Beta-lactam	1	0.19	cro-R	DQ302097
*catA1*	Phenicol	256	47.50	chl-R	V00622
*dfrA7*	Trimethoprim	257	47.68	tmp-R	JF806498
*qnrS1*	Quinolone	55	10.2	cip-R	AB187515
*strA*	Aminoglycoside	210	38.96	str-R	AF321551
*strB*	Aminoglycoside	210	38.96	str-R	M96392
*sul1*	Sulfonamide	257	47.68	sul-R	CP002151
*sul2*	Sulfonamide	265	49.17	sul-R	HQ840942, FJ197818, GQ421466
*tet*(A)	Tetracycline	51	9.46	tet-R	AJ517790
*tet*(B)	Tetracycline	46	8.53	tet-R	AF326777

a The table columns list each gene name, the antimicrobial class that it works against, the number of isolates that contained the gene, the percentage of isolates that contained the gene, the resulting resistance phenotype, and the NCBI gene accession number.

### Comparison of phenotypic and WGS-derived resistance profiles.

On the basis of the resistance genes identified for the five antimicrobial agents, a WGS resistance (WGS-res) profile was assigned and compared with the phenotypic profile of each isolate to evaluate the ability of the WGS approach to predict the resistance phenotype (Table 5). For all antimicrobial agents except ciprofloxacin, the two profiles corresponded at a level of 99% for the resistant isolates. In contrast, some susceptible isolates (*n* = 33) harbored resistance genes, which reduced the specificity of the method (≥91%). Three of them had truncated genes (considered inactive genes; Table 5), but the other 30 isolates had the complete coding sequences without any phenotypic resistance. This might suggest impairments (e.g., transcriptomic, translational, protein modification, etc.) in downstream steps of the resistance pathway or the presence of counteracting genes.

**TABLE 5:** Evaluation of the ability of WGS-res profiles to predict *S*. Typhi resistance phenotypes of our isolates^*a*^

Antimicrobial resistance category	Presence of gene(s) and WGS-res profile	No. of isolates with indicated phenotype		Sensitivity (%)^*b*^	Specificity (%)^*c*^
		Resistant	Susceptible		
Ampicillin resistance	Total	263	273		
	*bla*_TEM-1B_	262	7		
	Truncated *bla*_TEM-1B_	0	2		
	No *bla*_TEM-1B_	1	264		
	WGS-res profile: resistant	262	7	99.6	97.4
	WGS-res profile: susceptible	1	266		
					
Co-trimoxazole resistance^*d*^	Total	233	303		
	*dfrA7 *+* sul1 *+* sul2*	205^*e*^^,^^*i*^	4^*f*^^,^^*i*^		
	*dfrA7 *+* sul1* only	26	22^*g*^		
	*sul2* only	1^*e*^^,^^*i*^	55^*h*^^,^^*i*^		
	None of three	1	222		
	WGS-res profile: resistant	231	26	99.1	91.4
	WGS-res profile: susceptible	2	277		
					
Chloramphenicol resistance	Total	250	286		
	*catA1*	248^*j*^	7^*j*^		
	Truncated *catA1*	0	1^*j*^		
	No *catA1*	2	278		
	WGS-res profile: resistant	248	7	99.2	97.6
	WGS-res profile: susceptible	2	279		
					
Ceftriaxone resistance	Total	1	535		
	*bla*_CTX-M15_	1	0		
	No *bla*_CTX-M15_	0	535		
	WGS-res profile: resistant	1	0	100.0	NA
	WGS-res profile: susceptible	0	535		

a Four antimicrobials were considered (ampicillin, co-trimoxazole, chloramphenicol, and ceftriaxone); resistance to these agents is caused mainly by acquisition of resistance genes.

b Sensitivity data represent proportions of isolates identified as phenotypically resistant by the WGS-res profile.

c Specificity data represent proportions of isolates identified as phenotypically susceptible by the WGS-res profile.

d For co-trimoxazole (sxt), we considered the presence of *dfrA7*, plus *sul1* and/or *sul2* genes to exert the resistance (R) phenotype.

e A total of 206 detected *sul2* genes matched three different GenBank IDs: FJ197818 (*n* = 74), GQ421466 (*n* = 1), and HQ840942 (*n* = 131).

f Of the four *sul2* genes, two matched FJ197818 and two HQ840942. One *sul1* gene had unreliable bases (N) in its sequence; that result was considered a sequencing error, and the complete sequence was used in calculations.

g One *sul1* gene had unreliable bases (N) in its sequence; that result was considered a sequencing error, and the complete sequence was used in calculations.

h A total of 54 genes matched GQ421466 and one HQ840942.

i Only *sul2* genes that matched HQ840942 had complete sequences. Genes that matched FJ197818 and GQ421466 were either truncated or mutated.

j All *catA1* gene sequence had one silent mutation in amino acid 195 (lysine) (CTG→TTG).

On the other hand, for isolates with resistant phenotypes but susceptible WGS res-profiles, we screened for mutations in genes with efflux pump or membrane permeability functions (see Table S1 in the supplemental material). However, no relevant patterns were detected for AMR.

10.1128/mBio.02112-18.8 TABLE S1 List and characteristics of detected genes with efflux pump and membrane permeability activity. Download Table S1, DOCX file, 0.01 MB. Copyright © 2018 Tanmoy et al. 2018 Tanmoy et al. This content is distributed under the terms of the Creative Commons Attribution 4.0 International license.

### Ciprofloxacin resistance, background mutations, and genotypes.

**FIG 4:**
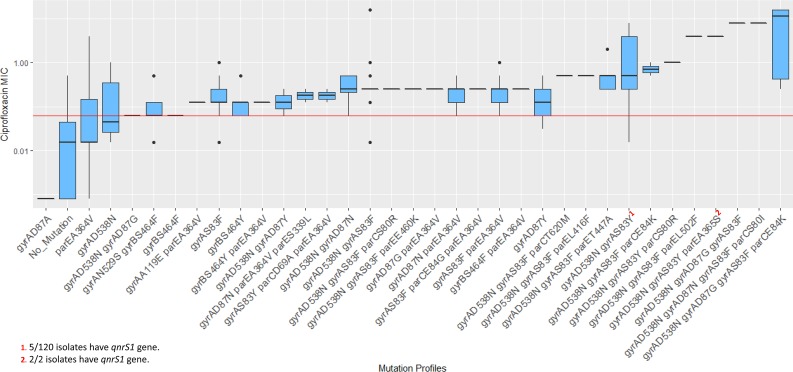
Mutation profiles detected in genes associated with ciprofloxacin resistance in our isolates and correlation with ciprofloxacin MIC. The horizontal red line indicates the threshold MIC level (0.06 µg/ml) of resistance (according to EUCAST v8.0).

**FIG 5:**
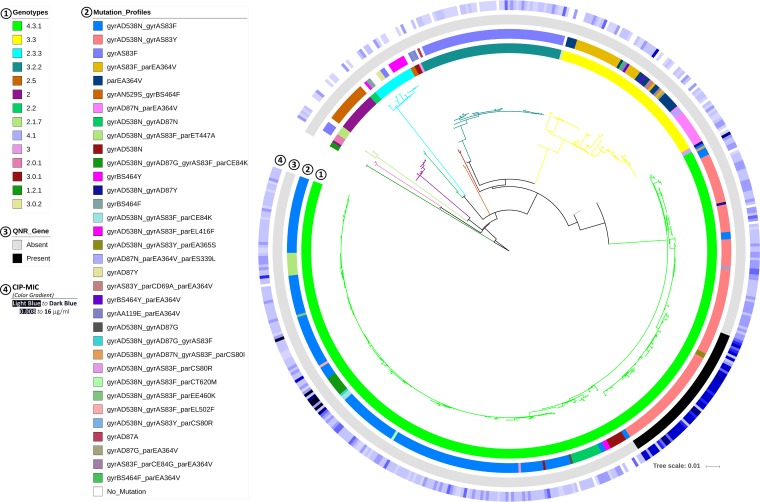
Comparison of mutation profiles and presence of *qnr* genes with the level of ciprofloxacin resistance (cip MIC) and genotypes in a wgSNP-MLT. No singleton was considered in the consensus SNP data. The tree is colored by genotype. Circles around the tree are numbered and colored based on the data points shown.

The resistance gene analysis identified the *qnrS1* gene in 55 isolates (Table 4) and detected a number of different mutations (*n* = 24) in the *gyrA* and *gyrB* genes encoding DNA gyrase and in the topoisomerase IV enzyme *parC* and *parE* genes (Table 6, columns 1 to 3). The most prevalent mutation was *gyrA* D538N (*n* = 352), followed by *gyrA* S83F (*n* = 299), *gyrA* S83Y (*n* = 125), and *parE* A364V (*n* = 69). On the basis of mutations in *gyrA*/*B* and *parC*/*E* genes, 34 cip-mutation profiles were generated and compared with the ciprofloxacin MIC of each isolate (Table 6, columns 4 and 5) (Fig. 4 and 5). All of the profiles, apart from *gyrA* D538N and *parE* A364V, were associated with resistance (MIC > 0.06 µg/ml). Two different profiles with triple mutations (*gyrA*_D87G_ *gyrA*_S83F_ *parC*_E84K_ for eight isolates and *gyrA*_D87N_ *gyrA*_S83F_ *parC*_S80I_ for one isolate) had median MICs of ≥8.0 µg/ml (Fig. 5) (Table 6, columns 4 and 5). Profiles with *qnr* genes also had MICs of ≥1.0 µg/ml (Fig. 4 and 5). No mutations were present in 18 isolates, but 3 of them showed resistance to ciprofloxacin (MIC of 0.25 to 0.5 µg/ml; Fig. 4).

**TABLE 6:** Mutations detected in DNA gyrase (*gyrA* and *gyrB* genes) and topoisomerase IV (*parC* and *parE* genes) individually, and combined mutation profiles based on them

Gene	Mutation	No. of mutations	Mutation combination (profile)	No. of mutation profiles
*gyrA*	D538N	352	*gyrA*-D538N, *gyrA*-S83F	179
	S83F	299	*gyrA*-D538N, *gyrA*-S83Y	120
	S83Y	125	*gyrA*-S83F	66
	D87N	30	*gyrA*-S83F, *parE*-A364V	26
	N529S	17	*parE*-A364V	18
	D87G	11	*gyrA*-N529S, *gyrB*-S464F	17
	D87Y	4	*gyrA*-D87N, *parE*-A364V	15
	A119E	1	*gyrA*-D538N, *gyrA*-D87N	12
	D87A	1	*gyrA*-D538N, *gyrA*-S83F, *parE*-T447A	9
*gyrB*	S464F	21	*gyrA*-D538N	8
	S464Y	10	*gyrA*-D538N, *gyrA*-D87G, *gyrA*-S83F, *parC*-E84K	8
*parC*	E84K	10	*gyrB*-S464Y	8
	S80R	2	*gyrB*-S464F	3
	D69A	2	*gyrA*-D538N, *gyrA*-D87Y	2
	T620M	1	*gyrB*-S464Y, *parE*-A364V	2
	E84G	1	*gyrA*-D87Y	2
	S80I	1	*gyrA*-S83Y, *parC*-D69A, *parE*-A364V	2
*parE*	A364V	69	*gyrA*-D538N, *gyrA*-S83F, *parE*-L416F	2
	T447A	9	*gyrA*-D87N, *parE*-A364V, *parE*-S339L	2
	L416F	2	*gyrA*-D538N, *gyrA*-S83Y, *parE*-A365S	2
	S339L	2	*gyrA*-D538N, *gyrA*-S83F, *parC*-E84K	2
	A365S	2	Other combination pattern (one isolate for each)	13
	L502F	1	No mutation	18
	E460K	1		
Total				536

Mutations in codon 83 of *gyrA* (S83F and S83Y) were the most prevalent among our ciprofloxacin-resistant isolates (411/467; 88%) (Table 6, columns 1 to 3). The S83Y mutation (122/411) was closely associated with genotype 4.3.1 in Bangladesh (123/125; 98%) and was present in 96% of our H58 lineage Bd isolates (104/108; Fig. 3). In contrast, 89% of our lineage Ia isolates had the S83F mutation (198/223) and exhibited lower mean (0.74 versus 1.71 µg/ml) and median (0.25 versus 0.5 µg/ml) ciprofloxacin MIC values than the lineage Bd isolates. The latter lineage also displayed more divergence than lineage I in Bangladesh (Fig. 3; mean pairwise distances, 12.8 versus 11.2). The root of lineage Bd contained isolates detected at earlier time points (1999 to 2004) and formed a noticeable subclade at the tip composed of isolates (*n* = 55) collected from 2006 onward (Fig. 3; see also Fig. S2 and S3). In addition, this small subclade had the universal presence of the *qnr* gene and MIC values of ≥1.0 µg/ml (Fig. 3). This small, *qnr*-specific subclade within lineage Bd can be defined by SNPs at nucleotide positions 1253109 (T→G), 2385340 (A→G), 2676540 (A→T), and 2688285 (C→T) of the CT18 reference genome (and is referred to as sublineage Bdq in the rest of this article). Comparison with other lineages in our cohort of isolates from Bangladesh revealed that sublineage Bdq had a very high median ciprofloxacin MIC (4 µg/ml; Fig. S4) and low divergence (mean pairwise distance, 8.4). Many isolates from Pakistan in lineage Ia also contained *qnr* genes (Fig. 3) but showed no specific divergence pattern.

10.1128/mBio.02112-18.2 FIG S2 Annual distribution of genotypes in a wgSNP-derived MLT, showing the mutation profiles, *qnr* genes, MIC, and CIP resistance phenotypes of our isolates. No singleton was considered in the consensus SNP data. The tree is colored by year, and additional information is displayed in the different circles. Download FIG S2, TIF file, 4.2 MB. Copyright © 2018 Tanmoy et al. 2018 Tanmoy et al. This content is distributed under the terms of the Creative Commons Attribution 4.0 International license.

10.1128/mBio.02112-18.3 FIG S3 Annual distribution of dominant H58 sublineages in Bangladesh. Only sublineages Ia, Bdq, and Bd (except Bdq) are considered here. Download FIG S3, TIF file, 3.2 MB. Copyright © 2018 Tanmoy et al. 2018 Tanmoy et al. This content is distributed under the terms of the Creative Commons Attribution 4.0 International license.

10.1128/mBio.02112-18.4 FIG S4 Comparison of the (a) ciprofloxacin MIC and (b) ceftriaxone MIC for different H58 lineages of isolates from Bangladesh. Download FIG S4, TIF file, 2.7 MB. Copyright © 2018 Tanmoy et al. 2018 Tanmoy et al. This content is distributed under the terms of the Creative Commons Attribution 4.0 International license.

In total, 11 ciprofloxacin-resistant isolates (11/467; 2%) did not have any other mutation in DNA gyrase and topoisomerase IV genes, leading to an estimated sensitivity of 98% for the WGS method in correctly predicting ciprofloxacin resistance. In contrast, 36 of 69 ciprofloxacin-susceptible isolates had at least one mutation (not linked to a specific genotype) in one of these four genes (specificity = 52%).

### Comparison with neighboring countries.

All genotype 4.3.1 isolates from Bangladesh (this study), Nepal (surveillance in Kathmandu), and Pakistan (outbreak in Sindh) had the same *gyrA* D538N mutation (Fig. S5). Likewise, the *parE* A364V mutation was present in all genotype 3.3 isolates from Bangladesh (70/70) and Nepal (17/19) and in genotype 3.3.1 (3/3) isolates from Nepal. Genotype 2.0 isolates from Bangladesh also had the *gyrA* N529S mutation present (94%; 17/18). However, none of these mutations seemed to have any association with AMR (Fig. 4).

10.1128/mBio.02112-18.5 FIG S5 Association of specific mutations with different genotypes in a wgSNP-derived MLT. No singleton was considered in the consensus SNP data. Mutations such as *gyrA* S538N, *gyrA* N529S, and *parE* A364V are shown in comparison with genotypes 4.3.1, 2.0, and 3.3, respectively. All isolates from Bangladesh (our study), Pakistan, and Nepal are included. Download FIG S5, TIF file, 3.9 MB. Copyright © 2018 Tanmoy et al. 2018 Tanmoy et al. This content is distributed under the terms of the Creative Commons Attribution 4.0 International license.

Comparisons performed with the *bla*_CTX-M-15_ gene sequence of our ceftriaxone-resistant isolate revealed 92% coverage and 99% identity with the XDR isolate (GenBank accession no. LT906492.1) from the Pakistani outbreak (Table 7). In contrast, the *bla*_CTX-M-15_ gene from Bangladesh shared complete homology with the Klebsiella pneumoniae *bla*_CTX-M-15_ gene (FJ815436.1). A detailed comparison of our sequence data with the sequences of the isolates from Pakistan and Nepal is presented in Table 7.

**TABLE 7:** Comparison of the isolates from Bangladesh with isolates described in other studies from two neighboring countries^*a*^

Criterion	Result(s)		
	Bangladesh (present study)	Nepal (29)	Pakistan (12)
Sample source	Hospital surveillance (hospitalized and outpatient services)	Laboratory surveillance of typhoidal *Salmonella*	Outbreak
Timeline	1999–2013	2008–2016	November 2016–March 2017
No. of *S*. Typhi samples analyzed	536	198	100
Age limit	<18 yrs for hospitalized cases; no age limit for outpatient cases	<14 yrs	None
No. of MDR or XDR isolates	MDR, 206 (38%); XDR, none	MDR, 6 (0.03%); XDR, none	MDR, 89 (89%); XDR, 87 (87%)
No. of isolates with ciprofloxacin resistance	467 (87%)	171 (86%)	96 (96%)
No. of isolates with ceftriaxone resistance (cro-R)	1 (0.2%) (caused by *bla*_CTX-M15_)	None	88 (88%) (caused by *bla*_CTX-M15_) (another 12 cro-S isolates were selected for comparison)
Genotype of cro-R isolate(s)	3.3	NA	4.3.1 (lineage Ia)
Phenotype of cro-R isolate(s)	amp-R, cro-R	NA	XDR
*bla*_CTX-M15_ identity and coverage	92% coverage and 99% identity with gene sequence from Pakistan	NA	NA
No. of isolates with indicated dominant genotypes	4.3.1 (H58), 350 (65%); 3.3 (H1), 69 (13%); 3.2.2 (H1), 61 (11%)	4.3.1 (H58), 154 (78%); 3.3.0, 19 (10%)	4.3.1 (H58), 99 (99%)
No. of isolates with indicated dominant H58 lineage(s)	Ia, 223 (63% of H58); Bd, 108 (31% of H58)	I, 21 (10% of H58); II 133 (67% of H58)	Ia, 92 (92% of H58)
Local lineage(s) detected?	Yes; lineage Bd (108 isolates [31% of H58]) and sublineage Bdq (55 isolates [16% of H58])	Yes; local lineage II (no. of isolates not given)	No; a clone of lineage Ia with possible local origin
AMR details of local lineage	All sublineage Bdq contain *qnr* genes; 88% have cip-MIC ≥1 µg/ml (median, 4 µg/ml)	(a) Intermediate resistance to CIP; (b) no MDR; (c) contains *gyrA*-S83F mutations	Not a lineage but a clone of lineage Ia; predominantly XDR
Time of emergence for local lineages	See Fig. S3	Possibly after 2008	November 2016–present

a MDR, multidrug resistance, defined as co-occurring resistance to ampicillin, chloramphenicol, and co-trimoxazole; XDR, extensive drug resistance, defined as MDR plus resistance to ciprofloxacin and ceftriaxone.

## DISCUSSION

### *S*. Typhi multilocus sequence types and other genotypes in Bangladesh.

Genotyping and the phylogenetic inferences agreed with the genotyping framework interpretation (27) and showed genotype 4.3.1 (haplotype 58, H58) to be dominant among the isolates from Bangladesh (Table 1). This was no surprise, as this genotype possibly emerged from South Asia in the early 1990s and now dominates in regions of typhoid endemicity in the world (26, 31). The same genotype was also dominant among the isolates from Nepal and Pakistan (Table 7) (12, 29). On the other hand, classical MLST revealed only three sequence types (ST), with dominance of ST1 and ST2, which accords with global MLST report (32). There are 46 complete MLST types available for *S*. Typhi (33). Interestingly, the third most common MLST type in our data, ST2209, had a complete match with genotype 2.3.3 (100%; 18/18) (Fig. 1a). Isolates of this genotype from 2013 (*n* = 8) had the same mutation (*gyrB* S464Y). Five of 8 had a cip-resistant phenotype, which could indicate the beginning of new clonal dissemination (see Fig. S2 in the supplemental material).

Moreover, 99% (349/351) of all ST1 isolates from Bangladesh belonged to genotype 4.3.1 (Fig. 1a). This association was previously described in a small study involving 32 isolates (34). A phylogeographical report of *S*. Typhi included an estimate that divergence for genotype 4.3.1 commenced in the very late 1980s (26). However, the presence of ST1 could be detected before the 1980s (33, 35), as is likely the case for H58.

### Presence of genotype-specific mutations.

Isolates with genotype 4.3.1 from all three countries shared a common but as-yet-unreported mutation, *gyrA* D538N (nucleotide position 2332398 of the CT18 genome; Fig. S5). This mutation is not linked to ciprofloxacin resistance (Fig. 4 and 5) but could be crucial to the structure of the DNA gyrase enzyme, considering the associated change in the isoelectronic point (pI; D→N: 2.77 → 5.41) of the amino acid due to this mutation (36). Similar associations were also observed between genotype 2.0 and *gyrA* N529S (hydrophobicity, 3.47 → 1.83), and genotype 3.3 and *parE* A364V (hydrophobicity, 0.0 → −0.78; Fig. S5) (37). These mutations could have potential as markers to trace genotypes, especially the more prevalent genotypes such as 4.3.1 and 3.3.

### New H58 lineages with high-level ciprofloxacin resistance.

According to the published scheme that defines the different lineages of genotype 4.3.1 (H58) (26, 38), lineage Ia was dominant among the isolates from the recent Pakistan XDR outbreak, while most isolates from the Nepal surveillance belong to lineage II (Table 7). An undefined cluster within lineage II was also noticed among the Nepali isolates (Fig. 3), as has been described previously (12, 29). Among our isolates from Bangladesh, we found a new lineage of genotype 4.3.1 (H58), Bd (*n* = 108), which represented the second most dominant lineage after Ia (*n* = 223). This new lineage has decreased susceptibility to ciprofloxacin compared to lineage Ia (mean MIC, 1.71 versus 0.74 µg/ml). Ciprofloxacin MICs of >0.06 µg/ml are classified as resistant following the EUCAST guidelines. However, as the resistance breakpoint specified by the Clinical and Laboratory Standards Institute (CLSI) is 1 µg/ml, some strains could be classified as susceptible in countries that use the CLSI guidelines (12, 17, 29). Moreover, lineage Bd is probably of local origin, as it was absent in both neighboring countries (Fig. 3) and does not match the published SNP definition of lineage I or II (38). This local variant also had a higher pairwise distance in the SNP matrix (mean, 12.8 versus 11.2) than lineage Ia, suggesting a different pattern of divergence.

Remarkably, a sublineage of lineage Bd (Bdq; *n* = 55) showed increased resistance compared to other isolates from the same lineage, with median ciprofloxacin MICs of 4.0 µg/ml (mean MIC, 3.4 versus 0.4 µg/ml; Fig. S4). Sublineage Bdq predominantly carried *qnr* genes, in addition to *gyrA* mutations (Fig. 3 and 5), and showed more clonality than other lineages (mean pairwise distance, 8.4 versus 11.2 for Ia). Moreover, sublineage Bdq emerged recently, as all isolates were from 2006 onward, but became more prevalent after 2007 (Fig. S3). Therefore, antimicrobial treatment with fluoroquinolones of infections caused by sublineage Bdq may lead to failure.

A similar highly resistant lineage with triple mutations (*gyrA*_S83F_ *gyrA*_D87G_ *parC*_E84G_) but with no *qnr* genes was previously reported to cause failure of treatment with gatifloxacin in Nepal (28, 29). Our MLT also showed a small subclade (*n* = 8) inside lineage Ia for Bangladesh, with a triple mutation (*gyrA*_S83F_ *gyrA*_D87G_ *parC*_E84K_) and median ciprofloxacin MICs of 8.0 µg/ml (Fig. 4 and 5).

Notably, the number of lineage II isolates (*n* = 4) in Bangladesh was extremely low (Fig. 3), despite the dominance of this lineage in Nepal and India (26, 27, 29). The surveillance data from Nepal, which mostly describes the isolates from Kathmandu valley, showed a shifting pattern of H58 lineages (from lineage I to lineage II) over the years (29). Such a changing pattern is not observed in Bangladesh, probably because of relatively high prevalence and dominance of local lineages, such as the previously unreported lineage Bd. The Nepal surveillance also reported association of MDR with lineage I and of cip resistance with lineage II (29). However, no such association has been found for lineage I or lineage Bd in Bangladesh (see Data Set S1 in the supplemental material).

10.1128/mBio.02112-18.9 DATA SET S1 Summary of our samples sequenced in this study. Data points include the following: sample identifier (id), accession numbers, year of isolation, age, sex, antimicrobial susceptibility patterns (ampicillin, AMP; co-trimoxazole, SXT; chloramphenicol, CHL; ciprofloxacin, CIP; ceftriaxone, CRO), MDR (or non-MDR) status, phenotype, MLST type, genotype, H58 lineages and sublineages, quinolone resistance-determining region (QRDR) mutations (in *gyrA*/*B* and *parC*/*E* genes), and presence of resistance genes (*bla*_TEM-1B_, *catA1*, *dfrA7*, *sul1*, *sul2*, *qnrS1*, *strA*, *strB*, *tetA*, and *tetB*). Download Data Set S1, XLSX file, 0.1 MB. Copyright © 2018 Tanmoy et al. 2018 Tanmoy et al. This content is distributed under the terms of the Creative Commons Attribution 4.0 International license.

### wgSNP analysis suggests regional clonality of *S*. Typhi in Bangladesh.

The wgSNP analyses of our isolates generated 2,328 SNPs, revealing that the *S*. Typhi population in Bangladesh is highly clonal. However, addition of the isolates from Nepal (*n* = 198) and Pakistan (*n* = 100) increased the number of SNPs to 3,251 but decreased the number to 627 for genotype 4.3.1 isolates only (*n* = 603). As the filtering criteria remain the same, the number of SNPs for all Bangladesh isolates is relatively low compared to the global or multicountry context (25–27) but is similar to country-specific data. For example, 1,850 SNPs were detected in isolates from Thailand (*n* = 44) and 2,187 SNPs in isolates from Nepal (*n* = 198) (29, 39). The wgSNP-MLT data showed distinct differentiation of all genotypes, much like the data from the cgMLST-UPGMA tree, except the latter lacked clear inferences for different H58 lineages (Fig. 1b and c and Fig. S6). Genotype 1.2.1 mapped close to the root of the MLT, suggesting that this genotype is one of the oldest circulating types. Likewise, being the most distantly related, genotype 4.3.1 could be one of the more recent genotypes circulating in Bangladesh (Fig. 1c) and neighboring countries (Fig. 2).

10.1128/mBio.02112-18.6 FIG S6 Comparison of all isolates from Bangladesh in a cgMLST-derived UPGMA tree. The tree is colored according to genotype. Data points such as those corresponding to MLST type, lineage, and sublineage are indicated (by colors) in different circles around the tree. Download FIG S6, TIF file, 4.2 MB. Copyright © 2018 Tanmoy et al. 2018 Tanmoy et al. This content is distributed under the terms of the Creative Commons Attribution 4.0 International license.

### WGS predicts AMR phenotypes with high sensitivity.

The WGS-based resistance profiles showed >99% sensitivity and >91% specificity in describing the phenotypes (for amp, sxt, chl, and cro) of AMR isolates (Table 5). Remarkably, the *dfrA7* genes (involved in trimethoprim resistance) were always detected in the presence of the *sul1* gene (sulfonamide resistance) and never alone. Table 5). Similarly, *sul1* was never detected in the absence of *dfrA7.* Two isolates had discordant results, as we did not detect the concordant resistance genes in WGS analyses (Table 5). Repeating the antimicrobial susceptibility tests (ASTs) reconfirmed the resistant phenotype. Other resistance mechanisms, e.g., efflux pumps or membrane permeability changes, may be involved (40).

Ciprofloxacin resistance in 11 isolates with no mutation in DNA gyrase or topoisomerase IV genes (and no *qnr* genes) can suggest the presence of other mechanisms. Indeed, MDR bacteria can increase the expression of efflux pump genes, including *acrAB*, *acrEF* and *tolC* (through overexpression of *ramA* or repression of *acrR* genes). This enables the bacteria to expel fluoroquinolone molecules, resulting in ciprofloxacin resistance (40–43), as well as ampicillin or chloramphenicol resistance, even in the absence of *bla* or *catA* genes (44–46). On the other hand, isolates carrying a *bla* gene without the resistance phenotype could be the result of mutations in the promoter regions of outer membrane protein genes, such as the *ompC* gene, which facilitates penetration of beta-lactams through the outer membrane (47, 48). This could be the scenario for several susceptible isolates (*n* = 30) in our library that have the full-length resistance gene. However, transcriptomic or proteomic approaches may be required to further explore these possibilities.

### Different genotypic backgrounds of ceftriaxone resistance in Bangladesh and Pakistan.

The ceftriaxone-resistant (cro-R) strain from our library was isolated in 2000. The first report of a cro-R strain was published in 1999 (16). Interestingly, this isolate harbored the same extended-spectrum-beta-lactamase (ESBL) gene, *bla*_CTX-M-15_ (17, 49, 50), that caused the ceftriaxone-resistant phenotype in an ongoing typhoid outbreak in Pakistan (12). Other ESBL genes, including *bla*_CMY-2_ and *bla*_CTX-M-14_, have also been reported in relation with ceftriaxone resistance in other *Salmonella* species (51, 52) but never in *S*. Typhi. The sequence identity of *bla*_CTX-M-15_ between our isolate and the Pakistani isolates was 99%, with 92% coverage (Table 7). The resistance phenotype and genotype were also different from those of our isolate (Table 7). The Pakistani outbreak isolates formed a distinct cluster in the H58-specific MLT and showed high-level clonality (Fig. 3 and Fig. S1). In contrast, our ceftriaxone-resistant isolate had genotype 3.3, which suggests a different source and geographical origin. Moreover, no other ceftriaxone-resistant strains of genotype 3.3 have been reported from Bangladesh. We hypothesize that acquisition of the *bla*_CTX-M15_ gene might compromise the fitness of *S*. Typhi although as of now no data have been published in support of this. Also, no association with fitness has been found for ciprofloxacin resistance mutations in DNA gyrase genes (53). Therefore, the possibility of a global dissemination of these recently emerging variants cannot be excluded given the successful multicontinent spreading of its H58 ancestor (genotype 4.3.1).

This study had some limitations. The isolates from Pakistan are from a still-ongoing outbreak in Hyderabad and Karachi that started in 2016. The Nepal isolates are from a prospective surveillance in the area of Kathmandu valley and cover a period of 9 years (2008 to 2016). The collection of strains from Bangladesh was selected from a biobank of >3,000 strains recovered over a period of 15 years (1999 to 2013) from two different hospital settings in Dhaka. The majority (97%) of the isolates from Bangladesh are from children (<18 years old). Therefore, none of the collections cover the whole population in their respective countries. Also, there is no overlap of the isolate collection periods between Bangladesh and Pakistan. Country-to-country comparisons of the observed data may therefore be biased.

### Conclusion.

Our study demonstrated that WGS has high sensitivity and specificity for prediction of *S*. Typhi resistance phenotypes. However, this genomic method still lacks sensitivity and needs fine-tuning for the detection of ciprofloxacin resistance. We detected three different mutations associated with specific genotypes that could be used to develop genotype-specific tracking tools. We report a new, local variant of genotype 4.3.1, lineage Bd, which contains a recently emerged sublineage, Bdq, that exhibits a high level of ciprofloxacin resistance. A triple mutant variant (*gyrA*_S83F_ *gyrA*_D87G_ *parC*_E84K_) of lineage Ia with high ciprofloxacin resistance was also detected. A similar triple mutant variant of lineage II (*gyrA*_S83F_ *gyrA*_D87G_ *parC*_E84G_) has been reported from Nepal and possesses the same phenotype (28, 29). Our ceftriaxone-resistant isolate contains the *bla*_CTX-M-15_ gene but has a genotype and gene sequence different from those of the same gene of XDR *S*. Typhi strains from the Pakistan outbreak, defining a different ancestral origin. Thus, dissemination of this isolate throughout the region from a single point is therefore less likely. However, multiple independent genetic events in neighboring countries and possible subsequent dissemination enhance the risk of the global spread of these highly resistant clones.

The data presented in this study will add to the accumulating information, from Pakistan and Nepal in particular, concerning the increasing drug resistance of *S*. Typhi. The emergence of XDR *S*. Typhi is strongly compromising effective treatment of typhoid fever. The spread of these resistant lineages and their occurrence in various Asian countries emphasize the need to inform public health professionals and sensitize the global community. Measures to implement a two-pronged approach for typhoid control need to be accelerated (54, 55). Both short-term vaccine interventions for high-risk populations and long-term water and sanitation interventions will undoubtedly be the cornerstones of a global prevention plan to address control of typhoid fever.

## MATERIALS AND METHODS

### Isolate collection and antimicrobial susceptibility profiles.

All *S*. Typhi isolates used in this study were collected from the Child Health Research Foundation (CHRF) at the Department of Microbiology, Dhaka Shishu (Children) Hospital, in Dhaka, Bangladesh. The CHRF team has been preserving invasive *Salmonella* isolates since 1999 and maintained a biobank of >3,500 *S*. Typhi isolates, largely from children (<18 years of age). All strains were isolated from the blood of patients diagnosed with typhoid fever in two different settings: hospital inpatients (hospitalized), and out-patients attending the consultation facility (56). Clinical and epidemiological data were collected for all isolates collected from hospital inpatients.

We selected 539 *S*. Typhi isolates for this study; data were available for those isolates with respect to the date of isolation (1999 to 2013), hospital setting, and phenotypic resistance for five different antibiotics (ampicillin, chloramphenicol, co-trimoxazole, ciprofloxacin, and ceftriaxone). Age data were available for 85% (456/536) cases; among those cases, 97% (443/456) patients were <18 years of age, while 76% (345/456) were <5 years of age. We checked the identity of the isolates by the use of standard biochemical tests and *Salmonella* agglutinating antisera (Thermo Scientific, MA, USA). Antimicrobial susceptibility for ampicillin (amp), co-trimoxazole (sxt), and chloramphenicol (chl) was determined using the disk diffusion method (Oxoid, Thermo Scientific, MA, USA). Broth microdilution was used to determine the MIC values for ciprofloxacin (cip) and ceftriaxone (cro; Sigma-Aldrich, MO, USA). All zone diameter and MIC data were interpreted according to EUCAST v8.0 clinical breakpoints (57). Fig. S7 in the supplemental material shows the complete workflow. All sequence data have been submitted to the European Nucleotide Archive (ENA). Data Set S1 in the supplemental material summarizes relevant details of our isolates.

10.1128/mBio.02112-18.7 FIG S7 Workflow of our study from the biobank of >3,000 *S*. Typhi isolates to the WGS data analysis of 539 isolates. Download FIG S7, TIF file, 2 MB. Copyright © 2018 Tanmoy et al. 2018 Tanmoy et al. This content is distributed under the terms of the Creative Commons Attribution 4.0 International license.

### DNA extraction and whole-genome sequencing.

Isolates were grown on MacConkey agar (Oxoid) overnight, and the colonies were suspended in water. The QIAamp DNA minikit (Qiagen, Hilden, Germany) was used to extract DNA from the suspension on the same day. WGS was performed using an Illumina HiSeq 4000 platform (The Oxford Genomics Centre at the Wellcome Trust Centre for Human Genetics, Oxford, United Kingdom). One Salmonella Paratyphi isolate was also sequenced so that it could be included in comparative phylogenetic analysis (as an outgroup).

### Data quality check.

Sequence data quality was checked using FastQC v0.11.15 (58). We summarized all quality indicators using MultiQC v3 (59). If the summary revealed the presence of adapter sequences, they were removed using Trimmomatic v0.36 (60). KmerFinder was used to confirm the species of the strains (61, 62). Another tool, SeqSero, was used for WGS-based serotyping, to determine the *Salmonella* serovar of the isolates and confirm the wet-lab serotyping results (63).

### WGS data analyses with BioNumerics.

Adaptor-free fastq files were imported into BioNumerics version 7.6.2 (Applied Maths NV, Sint-Martens-Latem, Belgium) and analyzed via the use of the integrated Calculation Engine. For the comparison with isolates from neighboring countries, we used recently published WGS data on 100 *S*. Typhi isolates from Pakistan (12) and 198 *S*. Typhi isolates from Nepal (29). The Pakistan isolates were mostly from an ongoing outbreak of XDR *S*. Typhi, in Hyderabad and Karachi, Sindh, Pakistan, between November 2016 and March 2017 (12). In contrast, the Nepal isolates were part of a hospital-based enteric fever surveillance performed during 2008 to 2016, based on one of the large referral hospitals in Kathmandu Valley, namely, Patan Academy of Health Sciences (PAHS). (29).

Details of the quality control of the WGS data, mapping against the reference genome, filtering the SNPs, allele calling for cgMLST, detecting the presence of acquired resistance genes, and SNP-based genotyping are described in Text S1.

10.1128/mBio.02112-18.11 TEXT S1 Supplemental methods. Download Text S1, DOCX file, 0.01 MB. Copyright © 2018 Tanmoy et al. 2018 Tanmoy et al. This content is distributed under the terms of the Creative Commons Attribution 4.0 International license.

### Classical 7-locus MLST.

The complete sequences of seven loci (*aroC*, *dnaN*, *hemD*, *hisD*, *purE*, *sucA*, and *thrA*) were identified in extracted contigs. All sequences were matched with Enterobase (Achtman 7-gene MLST) (http://enterobase.warwick.ac.uk/species/index/senterica) to determine the classical MLST type of each isolate.

### Phylogenetic analyses.

We used RaxML v8.2.10 to build maximum likelihood phylogenetic trees (MLT) (65) on the basis of the alignment of 2,328 SNPs from 536 *S*. Typhi isolates in our study, 3,251 SNPs from 834 isolates in the comparisons with neighboring countries, and 627 SNPs from all 603 H58 isolates. Lineages for all H58 isolates were determined as previously described (38). We employed the generalized time-reversible model and a Gamma distribution to model site-specific rate variation (the GTRGAMMA in RaxML). Support for the MLT phylogeny was assessed via 100 bootstrap pseudoanalyses. The *S*. Paratyphi A strain from Bangladesh (Sample: 311189_229186) was included as an outgroup for tree rooting. All MLT and UPGMA trees were displayed and annotated using the iTOL6 online version (66). To compute the genetic distances between different groups (e.g., countries, H58 lineages, etc.), a pairwise SNP distance matrix was generated between isolates by computing the number of SNP loci at which pairs of isolates had discordant alleles. Median distances within or between groups were computed from this distance matrix.

Statistical analyses were performed using R v3.5 (64); the same application was used to generate the line graphs and box plots.

### Data availability.

All sequence data determined in work have been submitted to the European Nucleotide Archive (ENA) (study identifier [ID]: ERP109468).

10.1128/mBio.02112-18.10 DATA SET S2 Summary of detail quality parameters of whole-genome sequences in this study. Different quality parameters at the posttrimming and *de novo* assembly stages were added separately. Download Data Set S2, XLSX file, 0.1 MB. Copyright © 2018 Tanmoy et al. 2018 Tanmoy et al. This content is distributed under the terms of the Creative Commons Attribution 4.0 International license.
