# Correction for Tanmoy et al., “Salmonella enterica Serovar Typhi in Bangladesh: Exploration of Genomic Diversity and Antimicrobial Resistance”

**DOI:** 10.1128/mBio.01044-21

**Published:** 2021-05-26

**Authors:** Arif M. Tanmoy, Emilie Westeel, Katrien De Bruyne, Johan Goris, Alain Rajoharison, Mohammad S. I. Sajib, Alex van Belkum, Samir K. Saha, Florence Komurian-Pradel, Hubert P. Endtz

**Affiliations:** a Department of Medical Microbiology and Infectious Diseases, Erasmus University Medical Center, Rotterdam, the Netherlands; b Fondation Mérieux and Centre International de Recherche en Infectiologie (CIRI), INSERM, Lyon, France; c Child Health Research Foundation, Department of Microbiology, Dhaka Shishu Hospital, Dhaka, Bangladesh; d Applied Maths, Sint-Martens-Latem, Belgium; e Data Analytics Unit, bioMérieux, La Balme Les Grottes, France; f Bangladesh Institute of Child Health, Dhaka Shishu Hospital, Dhaka, Bangladesh

## AUTHOR CORRECTION

Volume 9, issue 6, e02112-18, 2018, https://doi.org/10.1128/mBio.02112-18. In Data Set S1, the Raw Data Accession and Experiment Accession numbers of 516 samples were interchanged. The numbers have been corrected, and the file has been replaced online.

In the main text, the isolates were referred to using the descriptor “sample” from Data Set S1, not any of the corrected accession numbers. Therefore, this change does not affect the main text of the article in any way. Nonetheless, we apologize to the readers for any inconvenience caused by this error.

