# Supplementary figures and images for: Salmonella enterica Serovar Typhi in Bangladesh: Exploration of Genomic Diversity and Antimicrobial Resistance
Source: mBio. 2018 Nov 13;9(6):e02112-18. doi: 10.1128/mBio.02112-18 (PMC6234861; doi:10.1128/mBio.02112-18)

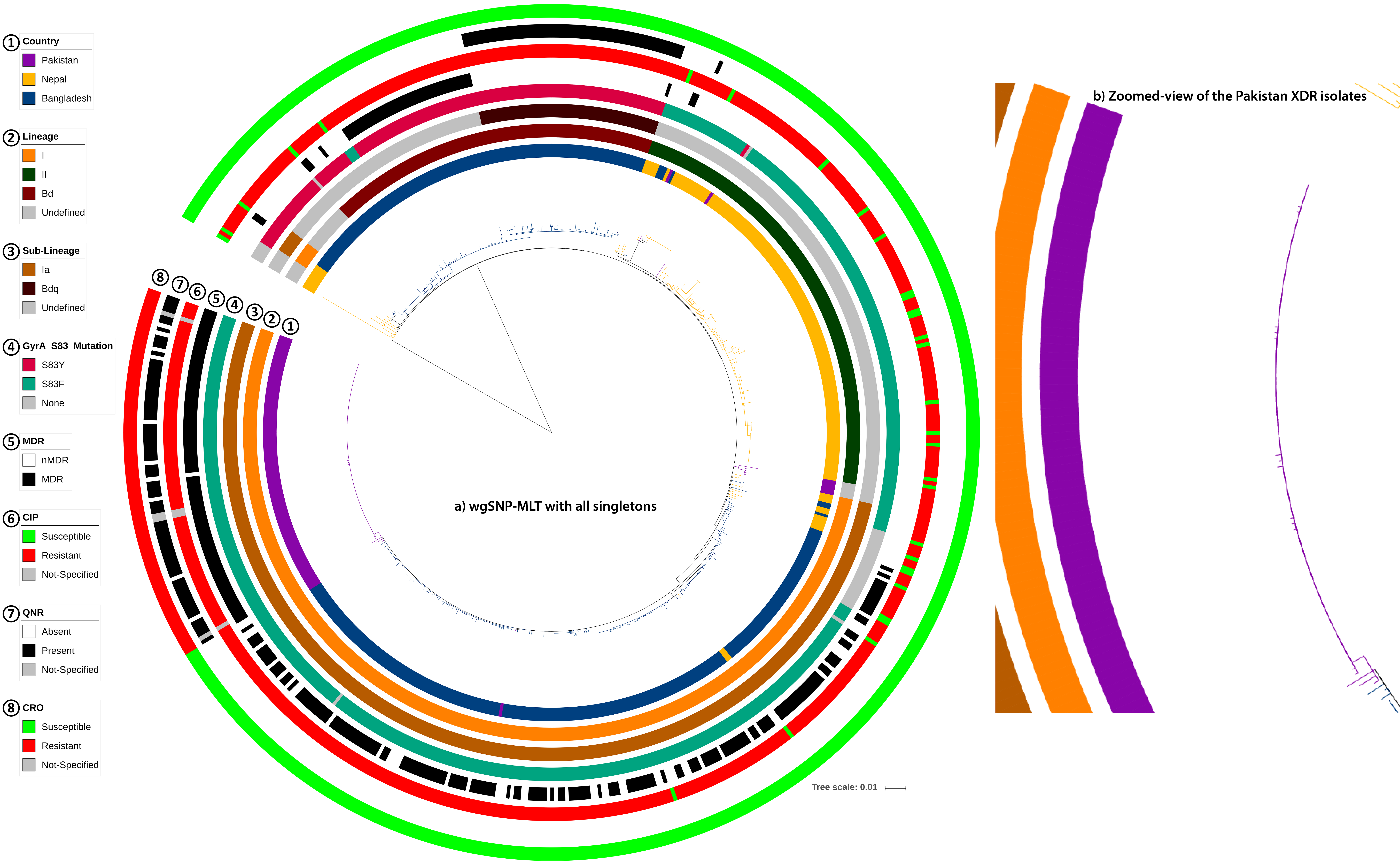

Supplement: FIG S1 [file mbo005184161sf1.tif]

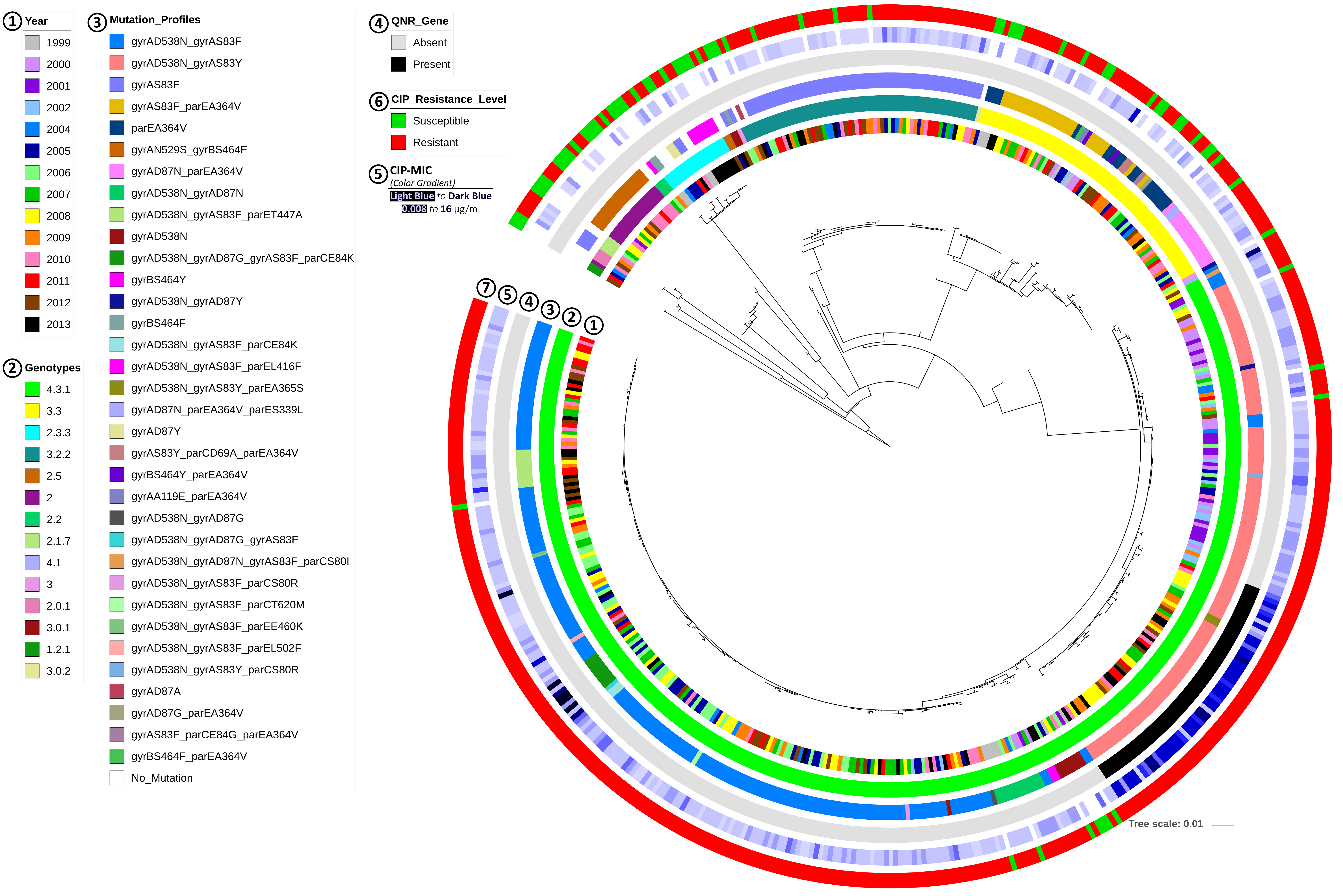

Supplement: FIG S2 [file mbo005184161sf2.tif]

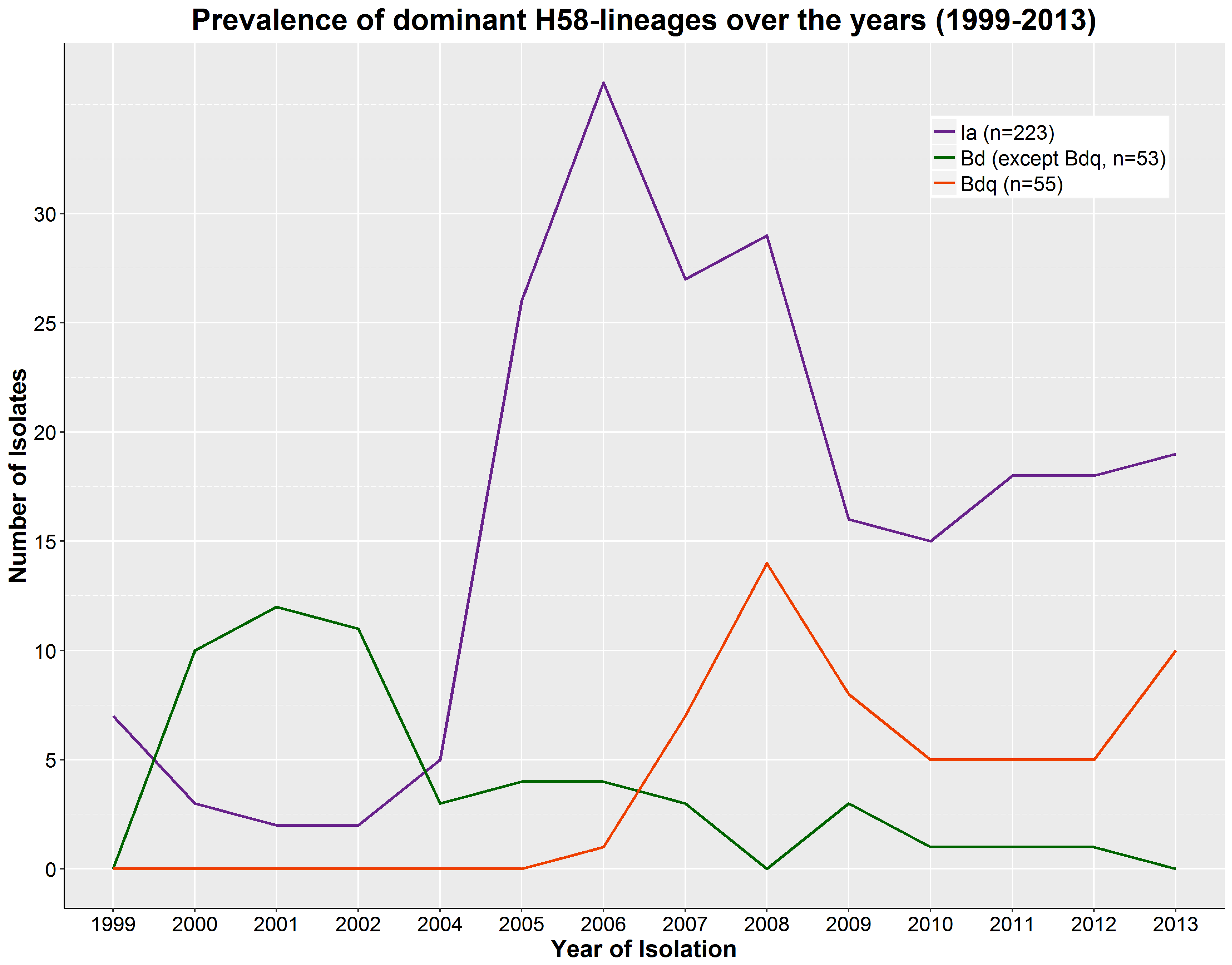

Supplement: FIG S3 [file mbo005184161sf3.tif]

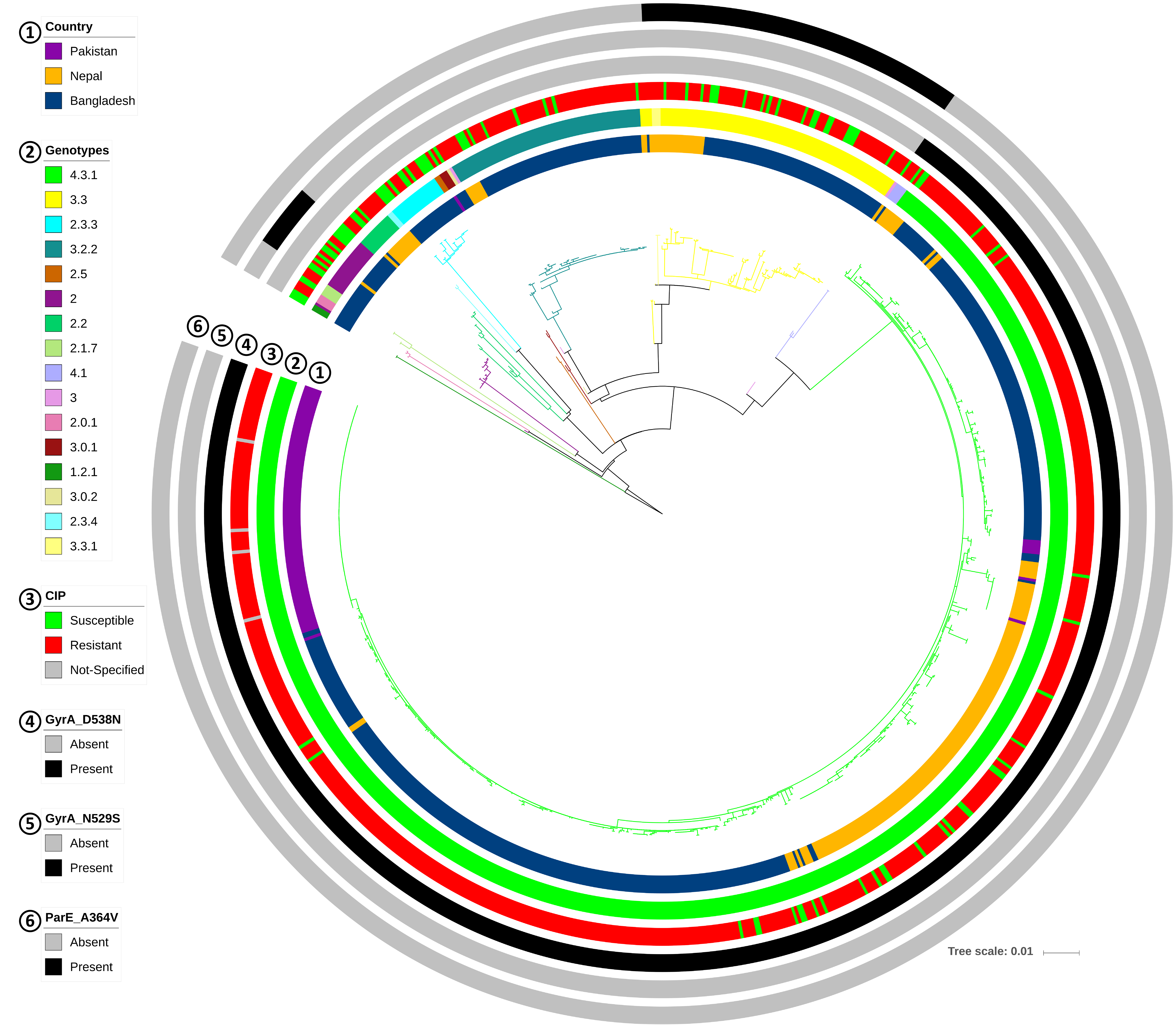

Supplement: FIG S5 [file mbo005184161sf5.tif]

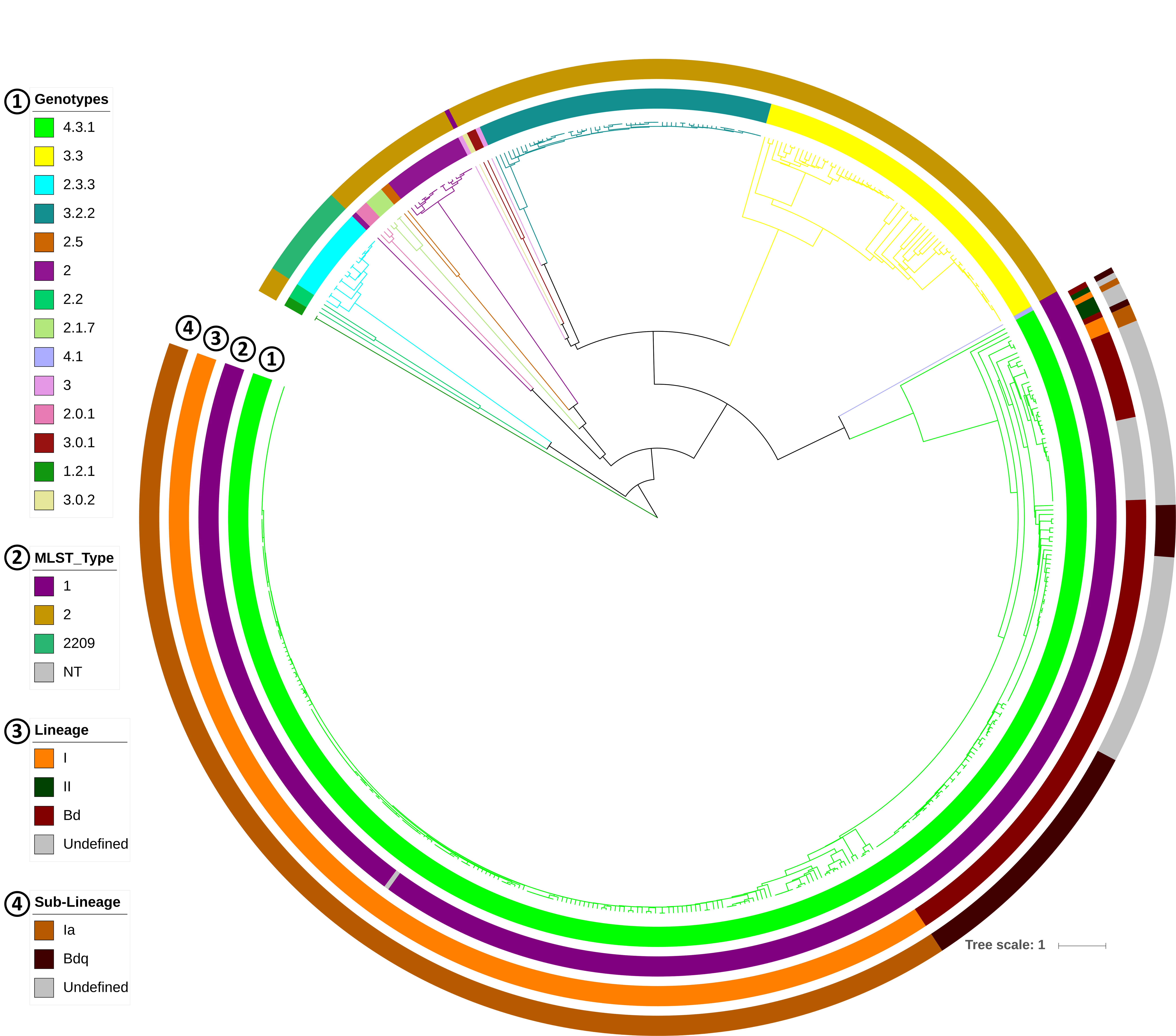

Supplement: FIG S6 [file mbo005184161sf6.tif]

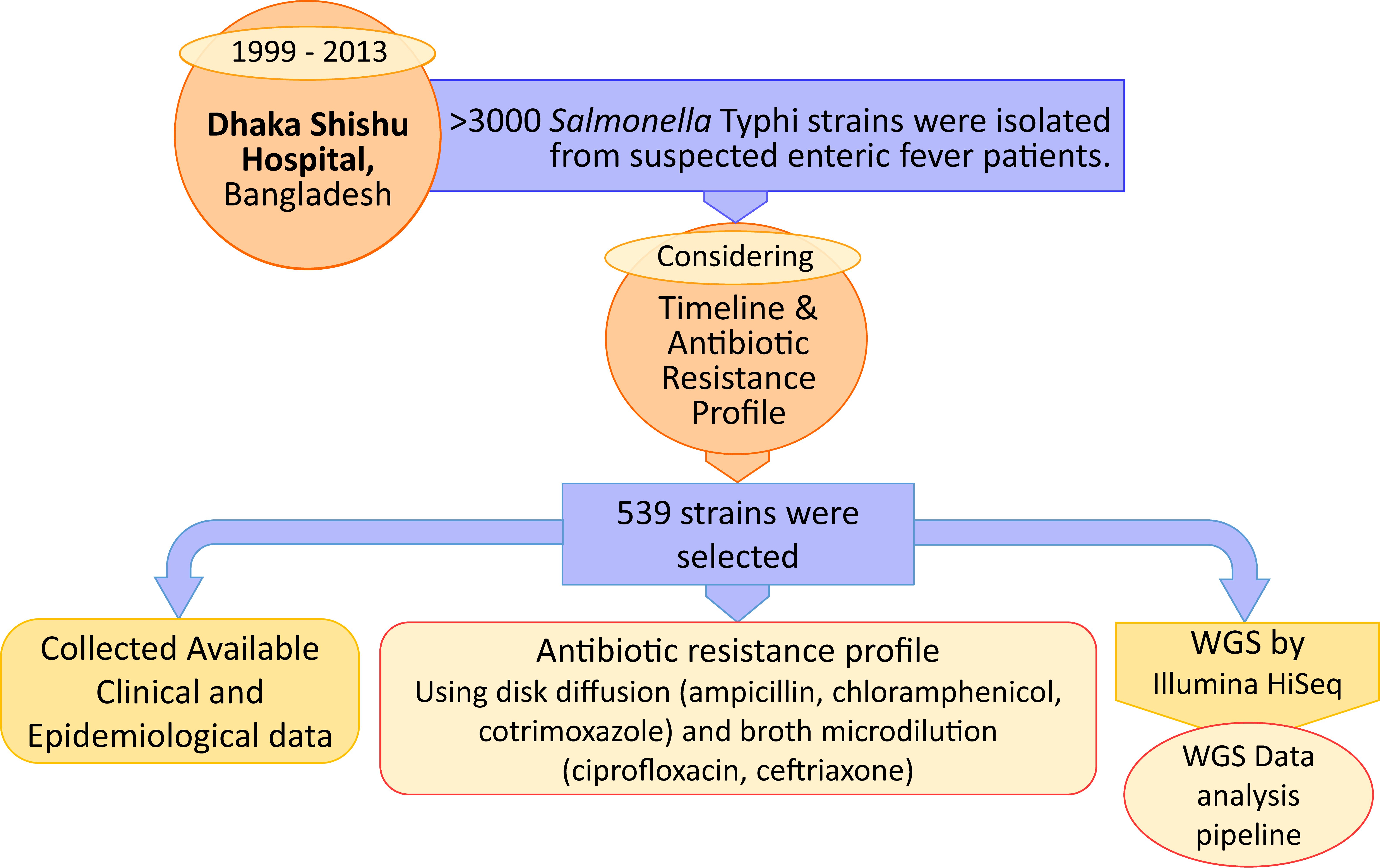

Supplement: FIG S7 [file mbo005184161sf7.tif]
