# Supplementary material for: Salmonella enterica Serovar Typhi in Bangladesh: Exploration of Genomic Diversity and Antimicrobial Resistance
Source: mBio. 2018 Nov 13;9(6):e02112-18. doi: 10.1128/mBio.02112-18 (PMC6234861; doi:10.1128/mBio.02112-18)
Supplement: TABLE S1 [file mbo005184161st1.docx]

**TABLE S1.** List and characteristics of detected genes with efflux pump and membrane permeability activity.

| Protein function | Name from CT18 (gene name) |
| --- | --- |
| Efflux pump | STY0414 |
| Acriflavin resistance protein | STY0519 (*acrB*), STY0520 (*acrA*), STY0521 (*acrR*), STY2719 (*acrD*), STY3569 (*acrE*), STY3570 (*acrF*) |
| Right origin-binding protein | STY4933 (*rob*) |
| Multiple antibiotic resistance protein | STY1541 (*marA*), STY1542 (*marB*), STY1539 (*marC*), STY1540 (*marR*) |
| Regulatory protein | STY4463 (*soxS*) |
| Outer membrane protein | STY3364 (*tolC*), STY2493 (*ompC*), STY1091 (*ompA*), STY1002 (*ompF*), STY0248 (*ompH*), STY1649 (*ompN*), STY4294 (*ompR*), STY2203 (*ompS1*), STY0872 (*ompX*) |
| Export membrane protein | STY0445 (*secD*), STY0446 (*secF*) |
| Integral membrane protein | STY0522 (*aefA*) |
| Membrane protein | STY0546 |
| Outer membrane porin L | STY3862 |
| Multidrug export protein | STY2940 (*emrA*), STY2941 (*emrB*), STY3981 (*emrD*) |
| Stress resistance protein | STY3452 |
