# Supplementary material for: Salmonella enterica Serovar Typhi in Bangladesh: Exploration of Genomic Diversity and Antimicrobial Resistance
Source: mBio. 2018 Nov 13;9(6):e02112-18. doi: 10.1128/mBio.02112-18 (PMC6234861; doi:10.1128/mBio.02112-18)
Supplement: TEXT S1 [file mbo005184161s1.docx]

*Salmonella* Typhi in Bangladesh: exploration of genomic diversity and antimicrobial resistance

Arif M. Tanmoy,^a,b,c^ Emilie Westeel,^b^ Katrien De Bruyne,^d^ Johan Goris,^d^ Alain Rajoharison,^b^ Mohammad S.I. Sajib,^c^ Alex van Belkum,^e^ Samir K. Saha,^c,f**^ Florence Komurian-Pradel,^b^* and Hubert P. Endtz^a,b^

^a^Erasmus University Medical Center, the Netherlands.

^b^Fondation Mérieux, Lyon, France.

^c^Child Health Research Foundation, Dhaka, Bangladesh.

^d^Applied Maths, Sint-Martens-Latem, Belgium.

^e^bioMérieux, La Balme Les Grottes, France.

^f^Bangladesh Institute of Child Health, Dhaka, Bangladesh.

*Corresponding author

**Co-corresponding author

A.M.T. and E.W. contributed equally to this article.

**Supplementary Methods**

**WGS data analyses with BioNumerics**

*Quality control of the data*

Quality control of the WGS data was performed by calculating the average read quality and read length statistics of the raw data. Automatic trimming was done following different parameters of raw data (fastq files), and includes- a) removal of reads if they are >20% shorter than the average read length or, has >10% less quality than the average quality and, b) shortening of reads if the quality at the edge gets >25% less than the average quality or, contains Ns. De novo assembly was performed using SPAdes v3.7 (67), including base-call correction by remapping the reads, as implemented within the Calculation engine of BioNumerics. Contigs were filtered by removing the smaller ones (<300 bases) and those with low coverage (cut-off value were automatically calculated in SPAdes with the option “cov-cutoff = auto”).

*Mapping against the reference genome*

For read alignment, paired-end Illumina reads from our *S.* Typhi isolates were mapped to the *S.* Typhi CT18 reference genome (NC_003198.1) using Bowtie2 (68) within the Calculation Engine. Next, to generate a consensus sequence, our parameters were- a) minimum coverage of three, including at least one base in each direction and, b) single base threshold of 0.75, a double base threshold of 0.85, a triple base threshold of 0.95 and a gap threshold was 0.5.

*Filtering the SNPs*

For SNP calling, those located in repeats, insertion sequences or phage regions were discarded. Only SNPs with a minimum total coverage of five reads, including at least one read in each direction, were considered. Positions with gaps or >1% unreliable or ambiguous bases were removed. Non-informative SNPs (i.e. same SNP present for all strains but not the reference sequence) and singleton SNPs (i.e. positions for which all but one sample had the same base call) were excluded. The required minimum distance between SNPs was 10 bases. Data set S2 summarizes the detail quality parameters of our isolates.

The same reference and filtering steps were followed for the comparison dataset of 834 *S.* Typhi (536 Bangladesh, 198 Nepal and 100 Pakistan) isolates. A separate analysis was performed for all 603 H58 isolates from all three countries (350 Bangladesh, 154 Nepal, and 99 Pakistan).

*Allele-calling for core-genome MLST (cgMLST)*

Assembly-free and assembly-based allele detection were used for the cgMLST analysis and performed using BioNumerics and the WGS tools plugin. The *Salmonella enterica* cgMLST scheme used in BioNumerics was based on the core genome MLST definition published on Enterobase (<https://enterobase.warwick.ac.uk/>). However, the pan-genomic scheme has been defined internally by Applied Maths and international coworkers, and is composed of 12,865 accessory loci, 3,002 core loci, and seven classical MLST loci (69).

Clustering of the cgMLST/SNP results was performed via the unweighted-pair group method using average (UPGMA) method, based on pairwise allele/SNP differences between isolates. UPGMA trees were constructed with a first priority rule of a maximum number of single-locus variants (weight: 10000), followed by a priority rule of a maximum number of double-locus variants (weight: 10).

*Detecting of the acquired resistance genes and gene mutations*

The presence of acquired resistance genes was assessed using the BioNumerics *Salmonella* plugin. Reads were mapped against a reference database based on available data sets from the Center for Genomic Epidemiology at Denmark Technical University (DTU), combined with private Applied Maths knowledge and expertise. The BLAST parameters for gene detection were set to 90% sequence identity and 60% sequence coverage. Mutations occurring in the DNA-gyrase *gyrA/B* and topoisomerase-IV *parC/E* genes with support from at least five reads (in any direction) were selected for ciprofloxacin resistance analysis.

For sequence extraction and alignment, each detected resistance gene was extracted from contigs using the sequence extraction tool available in BioNumerics. Next, all gene sequences and the reference sequence were aligned using the multiple alignment tool in BioNumerics, keeping the default parameters (open gap penalty, 100%; unit gap penalty, 0%; fast algorithm with minimum match sequence of 2; maximum gap number, 98). The same steps were followed for the genes related to efflux pumps and membrane permeability.

*wgSNP-based genotyping of Salmonella Typhi*

A *Salmonella* Typhi genotyping plugin based on the Genotyphi script (27) was used to determine the genotype of all 536 *S*. Typhi isolates from Bangladesh. We compared all genotypes with the previous haplotyping system (70), according to the description provided in Wong et al. (27).

**References**

27. Wong VK, Baker S, Connor TR, Pickard D, Page AJ, Dave J, Murphy N, Holliman R, Sefton A, Millar M. 2016. An extended genotyping framework for *Salmonella enterica* serovar Typhi, the cause of human typhoid. Nature Communications 7:12827.

67. Bankevich A, Nurk S, Antipov D, Gurevich AA, Dvorkin M, Kulikov AS, Lesin VM, Nikolenko SI, Pham S, Prjibelski AD. 2012. SPAdes: a new genome assembly algorithm and its applications to single-cell sequencing. Journal of Computational Biology 19:455-477.

68. Langmead B, Salzberg SL. 2012. Fast gapped-read alignment with Bowtie2. Nature Methods 9:357.

69. Achtman M, Wain J, Weill F-X, Nair S, Zhou Z, Sangal V, Krauland MG, Hale JL, Harbottle H, Uesbeck A. 2012. Multilocus sequence typing as a replacement for serotyping in *Salmonella enterica*. PLoS Pathogens 8:e1002776.

70. Roumagnac P, Weill F-X, Dolecek C, Baker S, Brisse S, Chinh NT, Le TAH, Acosta CJ, Farrar J, Dougan G. 2006. Evolutionary history of *Salmonella* typhi. Science 314:1301-1304.
